# Chemical Fingerprints of Emotional Body Odor

**DOI:** 10.3390/metabo10030084

**Published:** 2020-02-28

**Authors:** Monique A.M. Smeets, Egge A.E. Rosing, Doris M. Jacobs, Ewoud van Velzen, Jean H. Koek, Cor Blonk, Ilse Gortemaker, Marloes B. Eidhof, Benyamin Markovitch, Jasper de Groot, Gün R. Semin

**Affiliations:** 1 Unilever R&D Vlaardingen, Olivier van Noortlaan 120, 3133 AT Vlaardingen, The Netherlands; Ed.Rosing@unilever.com (E.A.E.R.); Doris.Jacobs@unilever.com (D.M.J.); ewoud-van.velzen@unilever.com (E.v.V.); jan.koek@unilever.com (J.H.K.); Cor.blonk@hccnet.nl (C.B.); i.gortemaker@upcmail.nl (I.G.); 2 Faculty of Social and Behavioural Sciences, Utrecht University, Heidelberglaan 1, 3584 CS Utrecht, The Netherlands; m.eidhof@reiniervanarkel.nl (M.B.E.); b.markovitch@umail.leidenuniv.nl (B.M.); j.h.b.degroot@uu.nl (J.d.G.); G.R.semin@uu.nl (G.R.S.); 3 William James Center for Research, ISPA-Instituto Universitário, 1149-041 Lisboa, Portugal

**Keywords:** body odor, chemical fingerprint, chemosignaling, gas chromatography-mass spectrometry, odor perception, pheromones, volatile organic compounds (VOCs), volatilome

## Abstract

Chemical communication is common among animals. In humans, the chemical basis of social communication has remained a black box, despite psychological and neural research showing distinctive physiological, behavioral, and neural consequences of body odors emitted during emotional states like fear and happiness. We used a multidisciplinary approach to examine whether molecular cues could be associated with an emotional state in the emitter. Our research revealed that the volatile molecules transmitting different emotions to perceivers also have objectively different chemical properties. Chemical analysis of underarm sweat collected from the same donors in fearful, happy, and emotionally neutral states was conducted using untargeted two-dimensional (GC×GC) coupled with time of flight (ToF) MS-based profiling. Based on the multivariate statistical analyses, we find that the pattern of chemical volatiles (N = 1655 peaks) associated with fearful state is clearly different from that associated with (pleasant) neutral state. Happy sweat is also significantly different from the other states, chemically, but shows a bipolar pattern of overlap with fearful as well as neutral state. Candidate chemical classes associated with emotional and neutral sweat have been identified, specifically, linear aldehydes, ketones, esters, and cyclic molecules (5 rings). This research constitutes a first step toward identifying the chemical fingerprints of emotion.

## 1. Introduction

The type of information human chemosignals (body odors) carry has been the subject of an increasing number of systematic investigations. The accumulating evidence from this research has highlighted the significant role that the neglected medium of olfactory communication (chemosignals) and the sensory system (the sense of smell) play in communication (e.g., [1,2,3]). This research has spanned the effects of body odors produced under different conditions of the donors, ranging from illness (e.g., [4]) to emotional states (e.g., fear: [5]). Despite the rapidly accumulating research on the effect of the information contained in body odors and its range, there is to our knowledge, no study that has analyzed the chemical composition of body odors and identified the distinctive chemical differences in the features of, for instance volatiles produced in a fearful versus happy state. This is precisely the focus of the current report. Below we first provide a very brief overview of the research on the information transmitted via human body odors. We then turn to the nature of the chemical composition of human odors and its characteristics that is the focus of the empirical work reported here.

“Communication occurs when a signal produced by an individual cause a reaction in another organism where both the signal and the reaction have been designed for these purposes” (Scott-Philips, 2015, p. 30; see [6]). Body odors have been found to serve precisely this signal function, and can range from bodily fluids such as sweat, to urine, tears, blood, and mother’s milk. The accumulating research to date has shown that they can signal information about the states and traits of individuals, such as emotional state [7,8,9,10], illness [4], gender [8,11,12], sexual preference [13,14], sexual arousal [15], kin [16], mate compatibility [17], menstrual phase [18] albeit some controversy on this subject [19,20], and personality traits such as extraversion [21]. The effects of human body odors do not appear to adhere to the strict definition of pheromones, as being single molecular compounds that activate very specific reactions (e.g., stereotyped behaviors, or developmental processes [22]). Therefore, the type of communication carried by human odors is increasingly referred to as chemosignaling [10,23].

Considerable progress has been made in our understanding of how emotions are chemosignaled, namely how emotion is transferred from one agent to another by means of body odors. Usually, these examinations used an experimental paradigm in which donors are exposed to emotion either inducing scenarios (e.g., film clips inducing fear, happiness, disgust states) or situations (e.g., stressful exams). During these sessions, the excreted odor is sampled from the armpit region. The collected samples are frozen and retained until the experimental session are thawed, and a recipient agent is then exposed to the odor. The experimental paradigms used a number of dependent measures depending on the type of emotional condition under which the donor produced the armpit odor and the predicted neural, cognitive, and behavioral consequences for the recipient. For example, in the case of fear odor one general question has been its consequences for increased vigilance (e.g., [24,25]), which has been measured by the caution manifested in a word choice task [24], the accuracy on an easy visual search task [25], the speed with which facial expressions were classified [26]. Another frequently used indicator of armpit sweat effects is facial muscle activity measurement (electromyography) indicative of facial emotion expressions [1,8,26,27,28]. Other assessments have focused on effects of chemical signals in relation to real-life settings such as influencing social judgment [29], and clinical performance of dental students [30]. Similarly, recordings of brain activity have been assessed with findings indicating higher event-related potentials at various time points (e.g., [31,32]), increased amygdala activity [33,34], and recruiting a wider network of brain regions in relation to empathy [35].

While it is clear that human chemosignals emitted by the sender are carriers of the information that is transmitted and received by the olfactory sensory system, the nature of the carriers remains a mystery. Body odor emanating from the underarm consists of a complex mixture of hundreds of chemical volatiles that is perceived holistically [36]. It is possible that a specific emotion signal is conveyed by a distinctive pattern of volatiles within an entire “package” of a volatile complex [36]. Thus, the dynamic nature of emotional states could mean that a distinctive set of volatiles are emitted or modified, generating in the brain a representation of that odor as being distinctively different from other such odors. An example not related to body odor would be the smell of coffee or wine, which are smelled by many people as distinctive and unique to the beverages that generate them, even though these smells emerge from chemical cocktails made up out of hundreds of volatile compounds [37]. We surmise that the emotion signal in body odor is conveyed by a pattern of chemical volatiles embedded in the larger mixture of volatiles, which in their combination may be regarded as characteristic of the emotional state experienced by the sender. It is unlikely that emotional specificity in body odor depends on a single (magical) molecule [2,22,23,38]. So rather than a single “fear” or “happiness” molecule, the more prevalent view about the nature of odor-print or odor signature is that there is a larger bouquet of chemicals shared across individuals that differ in terms of relative amounts [36]. This is why we introduced the concept of the emotional odor signals as patterns of chemical volatiles rather than single key molecules.

The current study was designed to examine the feasibility of emotion-specific odor signals or fingerprints in underarm sweat. First, we examined whether the patterns of volatile chemicals associated with underarm sweat samples from fearful, happy, and neutral donors, respectively, are distinctly different. Second, based on a first discrimination between chemical patterns, we tentatively identified the molecular classes associated with emotional and control sweat. An overview of volatile compounds emitted from the skin by Dormont et al. [39] lists “carboxylic acids of various chain lengths and derivative esters, aldehydes, alkanes, short chain alcohols, and some ketones” [39] with mainly alkanes and C6–C11 carboxylic acids being associated with the axillae. In another review, de Lacy Costello et al. [40] identified 532 named volatile compounds analyzed from skin secretions, although not in relation to the axillae specifically. The study reported here does not pretend to elucidate the definitive fingerprints of fear or happy emotion—the very existence of which might even be the topic of debate—yet it aims to progress our understanding of the chemical carriers of emotion communication by focusing on the primary candidate chemical classes, which may include any of the above.

We sampled underarm sweat from 24 male donors (for full details please see Methods) in a within-subjects design in which each donor participated in three emotion induction sessions (fear, happy, (pleasant) neutral) with a one-week interval between sessions during which the donors followed a protocolled regimen to keep contamination of sweat odor from odors related to other sources to a minimum. The emotion induction sessions involved watching pre-validated emotional movie clips for approximately half an hour. Following previous research [1,8,26,27,28], and for reasons explained in the Methods section, the order of sessions was not random, always starting with fear and ending with neutral. In the happy condition, as opposed to the fear and neutral condition, donors sat together in groups of 2, 3, or 4 to boost the positive experience. This was done because the co-presence of others heightens the positive emotional experience [41,42,43,44]. Self-report rating of emotion states was collected to check the effectiveness of the manipulation. The sweat was collected onto textile pads attached to the left and right underarm of pre-cleaned t-shirts. Textile pads were removed from the t-shirts and kept in sterile vacuum packs at temperatures of maximally −24 °C until the time of analysis.

Chemical analyses were conducted at Unilever R&D Vlaardingen, the Netherlands. All 144 textile pads were subjected to HeadSpace Sorptive Extraction combined with Thermal Desorption, followed by separation and detection using GC×GCMS. The Statistical Compare software feature within ChromaTOF^®^ was subsequently used to perform peak alignment between all samples. The resulting mass/retention time (MS/RT) peak table (3796 peaks) was exported and used for further multivariate statistical analysis. After data clean-up, 1655 volatiles were retained and subjected to principal component analysis (PCA), followed by partial least squares (using non-normalized, Pareto scaling, 7-fold cross-validation, and permutation testing) to identify statistically significant peaks testing three models: Fear vs. Neutral, Happy vs. Neutral, and Happy vs. Fear. The analysis focuses on commonalities of combined variables within a group and how they differ between the groups. Box plots were generated by using a statistical program (JMP^®^) for identifying and selecting volatiles showing significant differences.

We deliberately chose a conservative analysis that reveals only the strongest discriminators. As we did not know in advance which chemical features are associated with emotion signals, we believe than a comprehensive untargeted screening approach is appropriate.

Based on visual detection of a pattern of results corresponding to the Happy condition suggesting subclusters in the chemical data, cross-validated regularized logistic regression was conducted on the self-reported emotional states in all emotion conditions to explore whether these could account for these chemical subclusters. In other words, does the position donors occupy in the PCA score plot when in the Happy condition relate to their scores on the various self-reported emotional states?

## 2. Results

### 2.1. Emotion Manipulation

A total of 144 sweat pads were collected from the left and right underarm of 24 non-smoking Caucasian men (Mage = 22.42, SD = 3.54; range 19–34) during the three conditions Fear, Happy, and Neutral. 

**Figure 1:**
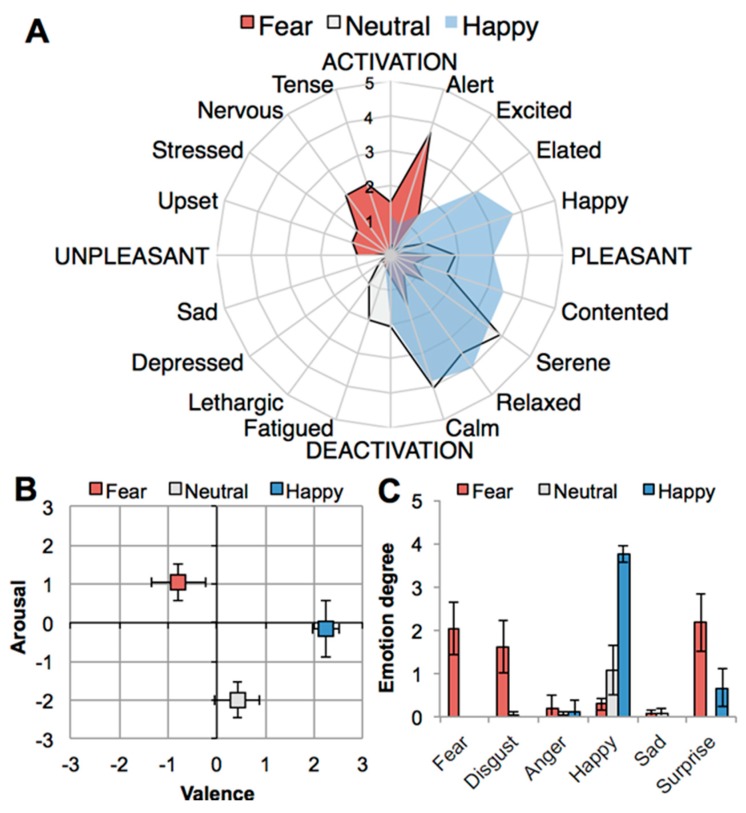
Effects of emotion induction (fear, neutral, happy) on self-reported emotional state: (**A**) Spider plot showing participants’ emotion profiles on affective circumplex [46]; (**B**) experienced core affect (valence, arousal) on affect grid [47]; (**C**) experienced discrete emotions. Error bars: ± 1 standard error of the mean (SEM).

Self-report data on emotional state were first subjected to a nonparametric Friedman test (degrees of freedom: 2, *n* = 24) to check for overall effects of condition (fear, happy, neutral) on self-report variables. Follow-up Wilcoxon signed-ranks tests were used to distinguish between specific pairs of conditions (fear-happy, fear-neutral, happy-neutral). Table 1 displays the results on effects of emotion condition on self-reported emotional state, demonstrating that emotion induction via movies was successful in eliciting the three target states (see also Figure 1): Happiness, χ²(2, *n* = 24) = 40.77, *p* < 0.001; Fear, χ²(2, *n* = 24) = 38,00 *p* < 0.001; and Calmness, χ²(2, *n* = 24) = 33.11, *p* < 0.001. Following [45] we refer to calmness as an indicator of a pleasant neutral state.

### 2.2. Chemical Analyses

**Figure 2:**
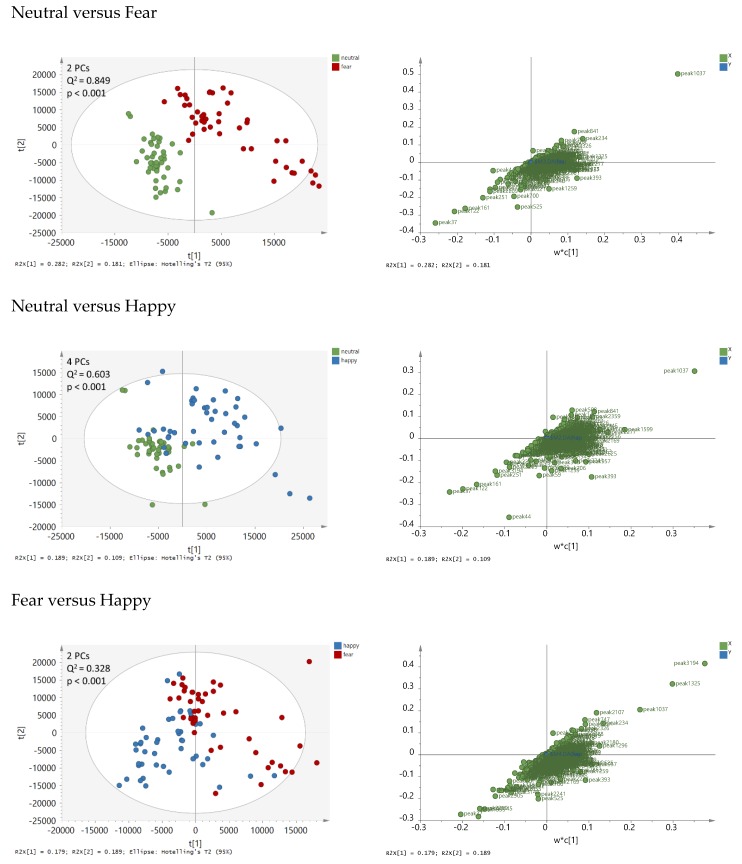
Partial least squares discriminant analysis (PLS-DA) score plots (left panels) and loading plots (right panels) based on 1655 peak areas and on two condition classes at a time: Neutral versus Fear (upper panels), Neutral versus Happy (middle panels), Fear versus Happy (lower panel). The first two principal components (PCs) t[1] and t[2] are displayed. The loading plots show weighted coefficients of the principal components. The data were not normalized and Pareto-scaled prior to analysis. Q^2^ values > 0.2 were statistically significant.

**Figure 3:**
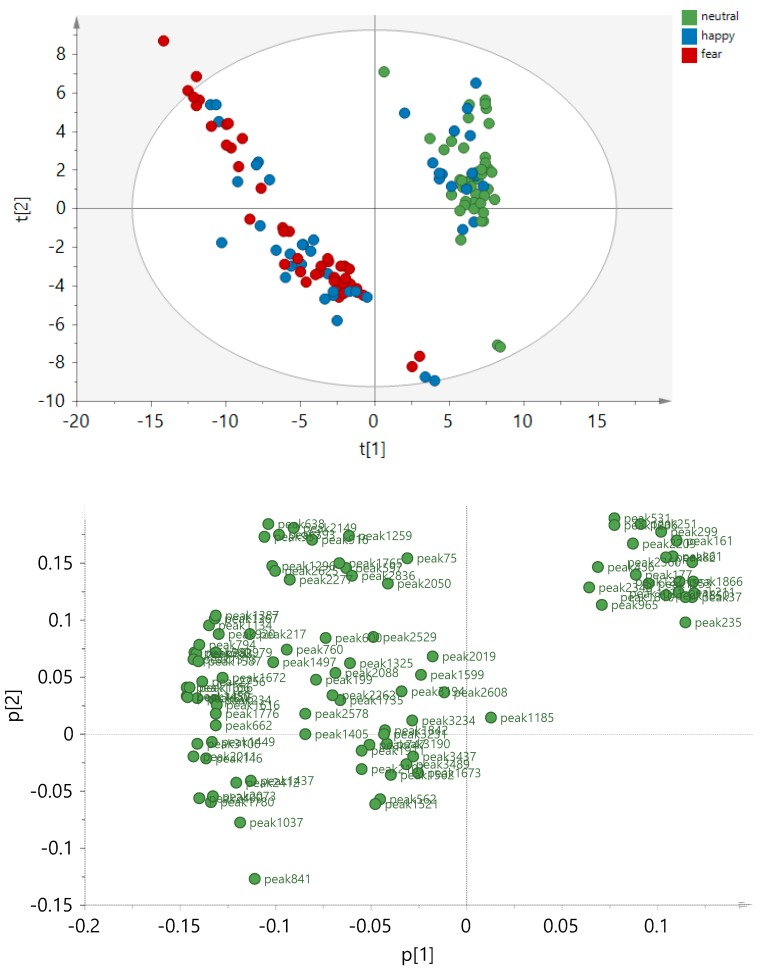
Principal component analysis (PCA) score plot based on 135 sweat samples and 94 peak areas that were significantly discriminating the condition groups in the PLS-DA models. The principal components t[1], t[2] are displayed, explaining R2X[1]= 0.437 and R2X[2] = 0.141 of the variances, respectively. The data were not normalized and Pareto scaling was applied.

To identify GC-MS peaks that have systematically higher or lower concentrations in one condition group than in the other, partial least squares discriminant analysis (PLS-DA) was performed on two condition classes, respectively. All three significant PLS-DA models, namely Neutral vs. Fear, Neutral vs Happy, and Fear vs. Happy sweat (Figure 2) were statistically significant. However, the strongest model was the Neutral vs. Fear (Q^2^ = 0.85), followed by Neutral vs. Happy (Q^2^ = 0.6). The Fear versus Happy model was weaker but still significant (Q^2^ = 0.33). From each model, the most discriminative peaks were identified, amounting to 94 peaks in total. They are listed in Table A1 (Appendix A). To visualize the relationships of the three condition groups to each other, principal component analysis (PCA) was performed based on these 94 peaks (Figure 3). Individual box plots for each of the peaks across the emotion conditions can be found in Supplementary Figure S1. From this analysis, it became evident, that Neutral and Fear are distinct classes while the happy condition showed a bipolar pattern, with a subcluster of data overlapping with the “fear” cluster, and another subcluster with the “neutral” cluster. An explanation for the two subclusters was explored via post-hoc testing below.

**Figure A1:**
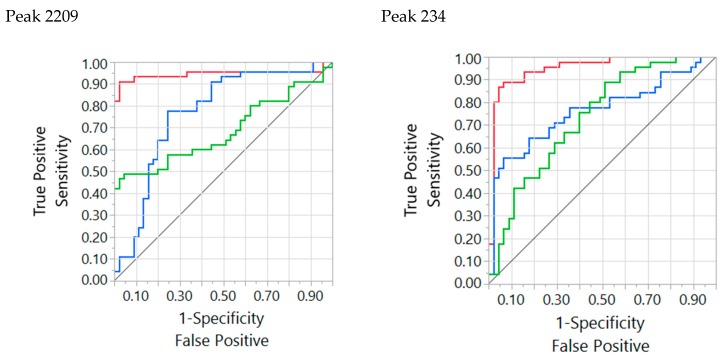
Receiver operating characteristic (ROC) curves of peak 2209 (left panel) and 234 (right panel) for the classifications Happy–Neutral (blue), Fear–Neutral (red), and Fear–Happy (green). For peak 2209 Neutral was the positive level for Happy–Neutral and Fear–Neutral, and Happy was the positive level for Fear–Happy. For 234 fear was the positive level for all three classifications.

Following the multivariate statistical analysis, the peaks found to be discriminative in the PLS-DA models were further analyzed using univariate statistics. For this, receiver operating characteristics (ROC) curves were calculated to measure the ability of each peak to discriminate between two types of emotion. In Figure A1 (Appendix A) two examples are depicted showing that peaks 2209 and 234 clearly discriminated Fear from Neutral. In contrast, the discriminative ability of these peaks was less when comparing Happy and Neutral, or Fear and Happy.

**Figure 4:**
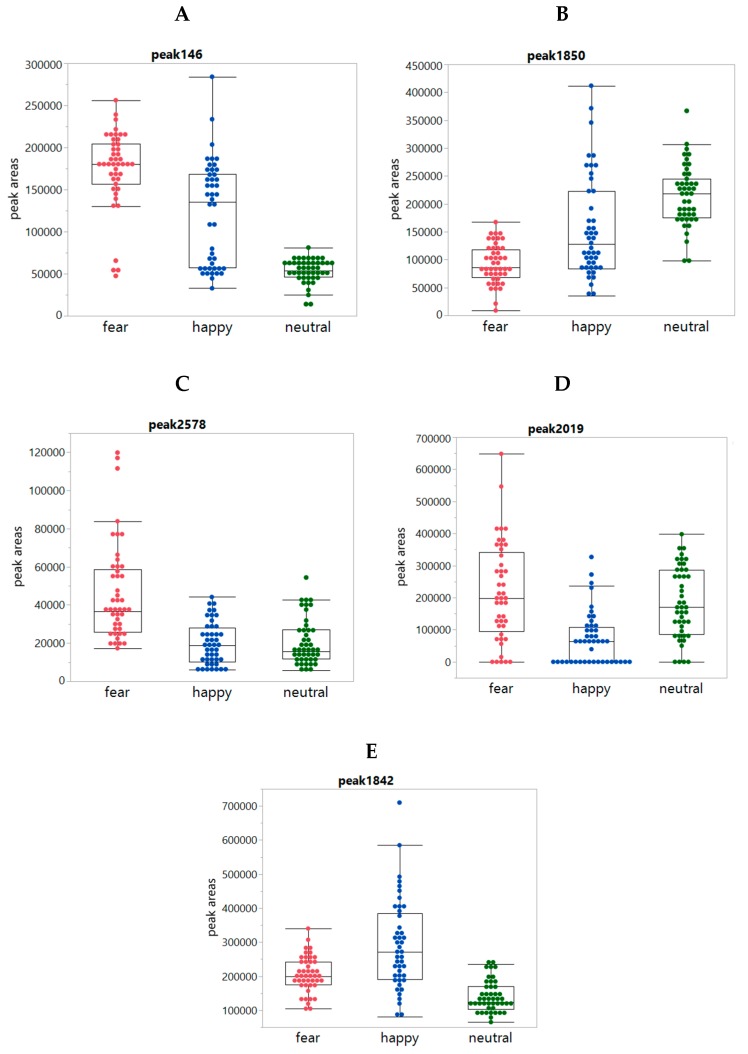
Box plots showing individual peaks per condition class: Panels (**A**–**E**) show the boxplots of the peaks 146, 1850, 2578, 2019, and 1842, respectively, per condition for each of the 24 panelists as observed in underarm sweat both left and right (so 48 datapoints for each of the three conditions in each graph).

**Figure A2:**
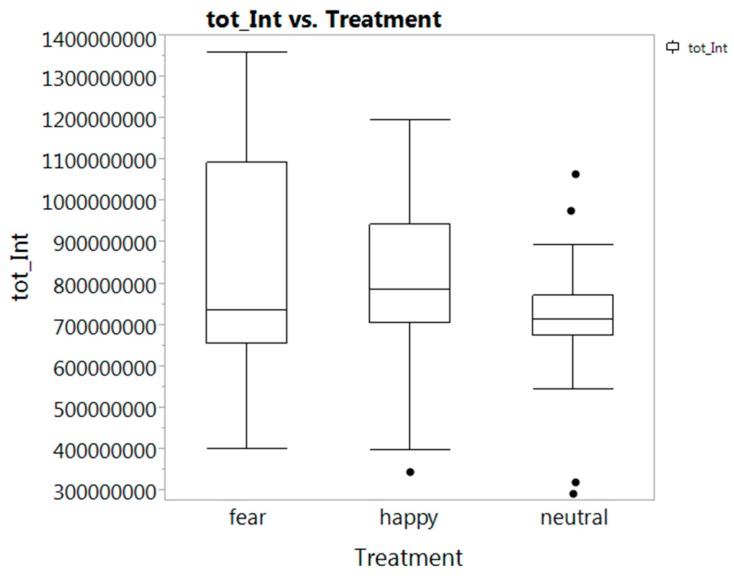
Box plot of three condition classes based on the sum of 1655 peak areas per sweat sample.

In addition, box plots comparing the emotion conditions on 94 statistically significant peaks were inspected, revealing clear differences between the conditions for 84 peaks. The remaining ten peaks showed only minor differences. From the 84 peaks, 36 peaks had increased intensities for both the Fear and Happy conditions when compared to Neutral (Figure 4A); 23 peaks had decreased intensities for both Fear and Happy conditions when compared to Neutral (Figure 4B); five peaks had increased intensities upon Fear when compared to Happy and Neutral (Figure 4C); 16 and 4 peaks were more increased (Figure 4D) and decreased (Figure 4E), respectively, in Happy when compared to Fear and Neutral. It is worth mentioning that most peak changes were not due to increased or decreased total sweat production, because there was (i) no significant difference in the total sweat production between the conditions (Figure A2, Appendix A) and (ii) no correlation between the peak intensities and the total intensities. Only for about 31 out of 94 peaks, the condition-dependent changes of the peak were related to increased total sweat production.

The data on 94 significant compounds were thereupon reviewed to investigate whether a logical chemical pattern could be detected to explain the chemical pattern of data. Two main types of chemical compound classes were identified: class 1 compounds (aldehydes/ketones) and class 2 compounds (esters and cyclic molecules (5 rings)). There was also a third class with 22 “happy compounds” that were mostly changed in the Happy when compared to Fear and Neutral. However, most of these compounds could not be identified or were identified as non-biological compounds, such as caprolactam. The Class 1 compounds (aldehydes/ketones) were high in the fear sweat and low in the neutral sweat condition and Class 2 compounds (esters and cyclic molecules) were high in the neutral condition and low in the fear sweat condition. The sweat of the subjects in the happy condition was either similar to the fear sweat or to the neutral sweat.

Further analysis on the subjects (*n* = 24) based on bipolar response in the happy condition resulted in two types of responses: response group 1 (*n* = 12) which we will refer to as sub-cluster HF (Happy overlapping with Fear) as it refers to individuals whose chemical patterns when in the happy condition overlap with the fear condition chemical patterns, and response group 2 (*n* = 8) which we will refer to as subcluster HN (Happy overlapping with Neutral) as their chemical patterns overlap with the neutral condition chemical patterns. This finding seems to suggest that the presumably emotional dimension that seems to be captured mostly by PC 1 and to some extent PC 2 (t[1] versus t[2] respectively in Figure 3) may be characterized by an increase in the linear aldehydes/ketones and a decrease in the esters and cyclic molecules (5 rings). That is, the high t[1](neutral both groups + subcluster HN) sweat in Figure 3 was high in esters and 5 rings, but low in linear aldehydes/ketones, while the low t[1] (fear both groups + subcluster HF) sweat was low in esters and 5 rings, but high in linear aldehydes/ketones (Table 2). More details about the members of the two chemical classes can be found in Table 3.

### 2.3. Exploring the Pattern of Bipolarity

Additional analyses were run to explore whether the bipolar pattern in the chemical data from the happy condition, referred to as HF and HN sub-clusters, can be explained by differences in emotional profile based on self-report, or on other factors. Figure 1A shows that profiles for self-reported emotional states for happy and neutral in particular, overlap, and hence it is possible that those individuals whose emotional state when happy overlapped to some extent with neutral, may have shown a correlated pattern for their chemical sweat odor profiles. We thereupon investigated whether the sub-clusters HF and HN differed in self-reported happiness. Happiness was virtually indistinguishable between the chemical sub-clusters, and very high on both (MHF = 3.79, SDHF = 0.42, MHN = 3.75, SDHN = 0.46, t(20) = 0.18, *p* = 0.856), so the difference in chemical configuration could not be explained by bimodality in happiness experienced by the donors. We thereupon investigated whether the bimodal pattern could be explained by systematic differences in emotional states other than happiness (see Table 1, Figure 1A). This turned out not to be the case. For these analyses and statistical approach we refer to Supplementary material S2A,B: Additional testing to explore bipolar pattern in happy chemical clusters.

## 3. Discussion

This study was conducted to explore the possibility of identifying chemical fingerprints of distinct emotional states in sweat odor sampled from the axillae of male donors in fear, happy, and neutral emotional states. Support was obtained for the notion of signal specificity in axillary sweat odor, as clusters of chemical data in a two-dimensional space spanning PC1 and PC2 obtained from PCA conducted on a matrix of 1655 chemical volatile peaks showed clearly distinctive clusters significantly separating fingerprints from fear versus neutral, as well as happy versus neutral, and happy versus fear. The distinction between the clusters showed up most clearly for the contrast fear versus neutral, while the pattern corresponding to the happy condition is best described as consisting of two subclusters, with one such cluster overlapping in chemical space with fear (HF), the other with neutral (HN). In general, the patterns are best described as Fear and HF being high in intensity of linear aldehydes and ketones, and low on esters and cyclic structures, and Neutral and HN showing the opposite pattern. Referring to the literature, the overview of chemical volatiles analyzed from the body compiled by de Lacy Costello et al. [40] as displayed in Figure 2 of their review [40] shows a large variety of chemical classes listing ten classes of chemical compounds for skin that include the four identified in our study, alongside others such as nitrogen- and sulfur-containing classes. Our analyses suggest that differences across emotion versus neutral sweat are most likely to be found in intensity differences of the aforementioned four chemical classes. Previously, using data mining techniques on a database of over 100 trace gas species, Williams et al. [48] had found differences in airborne chemicals emitted into the air of a cinema by audiences watching movies. Specific film events (i.e., “suspense” versus “comedy”) were associated with different levels of chemicals (such as acetone and isoprene) being emitted into the air. Furthermore, a recent publication by Preti et al. [49] comparing VOC composition of exhaled breath from individuals after validated stress induction to breath collected after a non-stressed control condition using GC/MS analytical techniques revealed three candidate biomarkers of stress: dimethyl sulfide and again, acetone, isoprene. It is not immediately clear how these results relate to our findings, as this would entail a comparison of stress-related volatiles in the oral cavity versus underarm, which are different environments. However, the results from both abovementioned studies support the notion of some specificity of volatile biomarkers relating to emotional state.

Previous reports on chemical analysis from odor arising from the axillary have also listed long-chain acids such as (E)-2-methyl-3-hexenoic-acid [50,51] as strong contributors to malodor. This compound, along with other typical compounds such hexanoic acid, butanoic acid, valeric acid, and 3-hydroxy-3-methylhexanoic acid (3H3MH), was picked up in our analyses as well but did not appear among the set of 94 compounds for which significant differences in peak intensity between the emotion conditions were found. This suggests that compounds previously identified as “smelly” are not necessarily also involved in chemosignalling of emotion.

In order to better understand the subclusters within the happy condition we examined the possibility of a relation to self-reported emotional state. However, we did not find support for a correspondence between these chemical clustering in space of the happy donors and their self-reported happiness or other emotional states. One could have expected a relation with arousal to emerge, as it is possible that some males experienced enhanced arousal in both the fear and happy states. This could have come about if the happy movie clips made them feel excited and invigorated in a way that corresponded to the arousal state resulting from the fear induction. Likewise, in other males the happy clips may have lowered the arousal levels comparable to the neutral condition because the happy clips had a soothing effect instead. However, this pattern did not come out. Self-reported arousal may not have been able to pick the proposed systematic relationship up. It would have been more appropriate to gage arousal with physiological endpoints such as heart rate and skin conductance.

Finally, we considered whether differences in movement across the conditions could account for the differences in patterns of volatiles. However, well-known indicators of energy metabolism like branched amino acids [52] and metabolites like acetoacetate and beta-hydroxybutyrate [53] are not volatile enough to detect with the used GC-MS technique.

Therefore, except for acetone which did not show up as different across conditions, neither acetoacetate nor beta-hydroxybutyrate were detected without further derivatization agents. However, assuming that the significant linear aldehydes and ketones we detected were correlated with movement, then the observed pattern across conditions does not seem to be entirely logical. While we encountered low levels of linear aldehydes and ketones during the emotionally neutral condition (as expected), these levels were high in the fear condition when participants did not move much. As noted earlier, in the happy condition 14 individuals showed a high response in terms of emitted volatiles whereas eight showed a low response. In conclusion, it is unclear when considering the present data whether differences in movement across the conditions caused the observed patterns of volatile emissions. Moreover, we would like to point out that rather than considering movement as a confound, we would consider movement (or lack thereof) as intrinsic to, or part of, the emotional states of interest. For future studies, it would be informative to include movement measures to explore the correlation between volatiles and movement in relation to emotional state.

There are a few limitations to our research: we used a fixed order of emotion induction for reasons that goes against the common practice of counterbalancing conditions. These reasons have been explained in the Methods section and are related to the inherent difficulty of inducing emotions in a laboratory setting knowing they are also influenced by pre-existing affective states [45]. Consequently, inducing fear during the first visit when cortisol levels are known to be high [54], served to maximize the chances of success at inducing fear. The self-reported levels of emotion from the donors in our study corroborated effective induction of fear, happiness, and a calm “pleasant neutral” state by following this fixed order.

To address the issue of fixed order, in a recently published study by our team [55] fear and pleasant-neutral were counterbalanced and sessions were held on the same day to simplify logistics. We found non-significant order effects on physiology and subjective ratings of fear. Counterbalancing also did not abolish the typical fear responses shown by receivers in a subsequent receiver study [56]. As this finding emerged after we had conducted our study, we recommend the use of counterbalanced designs in future research; however, in the present research, we wanted to use a cautious approach that mimicked prior research.

Another limitation may apply to the happy emotion induction condition. Here, happiness was induced while 2–4 donors were sitting together as opposed to being alone as was the case in the fear and neutral emotion induction conditions. The decision to do this was based on the desire to maximize the chances of success of happiness induction as the social presence of others can boost the positive experience, as pointed out earlier. The self-reported happiness/positive emotion ratings demonstrate that the happiness induction was indeed effective. However, the possibility remains that chemical volatiles associated with the body odor emanated during that session may have reflected “social” processes rather than “happy.” Data collected independent of the present study to address this issue are reported as Supplementary material S3. We conclude from the results of this study that effective emotion condition can emerge independently from the social presence or others suggesting that it is unlikely that this factor influenced the chemical composition of sweat as much as the experienced emotion.

Finally, and referring to Lenochova, Roberts, and Havlicek [57] who showed no differences in perceptual evaluations of sweat collected fresh versus after 4 months of freezing, we do not expect an effect of freezer storage time on sweat composition. In the present study, sweat was stored in the freezer at −24 °C directly after collection; all samples were collected over a one month period and shipped together to the analytical facility where they were stored at −80 °C and analyzed shortly after arrival. Thus, we conclude that the difference in composition of volatiles between emotional sweat clusters reflects the differences in emotional state induced in and reported by the donors as intended.

In previous research by our group, the emotional signal in the sweat volatiles was validated by the finding that receivers exposed to donors’ odor displayed a simulacrum in their facial expressions of the emotion experienced by the donors [1,8,25,26,27,28] The correspondence between donor and recipient could not be tested in the present study, because all sweat that was collected was used for the purpose of chemical analysis. Nevertheless, the evidence for such a correspondence has been documented in numerous studies. Notably, as a first step, differences in sweat composition correlating to emotional versus neutral state in sweat donors was confirmed.

Likewise, the biological activity of the extracted chemical characteristics for the different body orders can only be established if it were possible to artificially compose odors with the same properties that were extracted and show that these reproduce the reactions found earlier for the biological material collected (“Koch’s postulates”, see [22]). This is an avenue for future research.

As far as we know, this was the first study to investigate a systematic relation between emotional states and sweat odor emanated during such states. Our results underscore the likelihood of chemical fingerprints of emotion in sweat volatiles related to the emotional state. Specifically these differences emerge in the chemical compound classes of linear aldehydes, ketones, esters, and 5 rings as candidate key compounds. The distinctive fingerprints reported here support the possibility of emotion-specific signals excreted by a sender enabling a social communication with a receiver. Previous research by our group suggests that this chemical communication can elicit emotional contagion between the sender and receiver putting both individuals on the same page. These results again demonstrate the remarkable social functionality (see also Stevenson [58]) of the sense of smell, being not just limited to animals, but extending to humans [59].

In view of the exploratory nature of this research, we used a comprehensive, nontargeted screening approach. Using this conservative method, only the strongest discriminators emerged. As a next step, we call on others to conduct separate studies to replicate these results and validate our variables in a concerted effort of unravelling the signal of happiness and fear.

## 4. Materials and Methods

### 4.1. Emotion Induction and Sweat Collection from Donors

Twenty-four non-smoking Caucasian men (M_age_ = 22.52 years, SD = 3.50) were recruited at Utrecht University in 2014 to participate in this study. Participants were included if they reported experiencing fear during horror movies and not scoring extremely high on neuroticism or psychoticism. All men provided written informed consent to follow a regimen to prevent odor contamination of underarm sweat which prescribed adherence to a diet, the use of fragrance-free personal care products, and restriction of certain behaviors for 2 days prior to each sweat sampling session, and to donate sweat in three consecutive sessions (fear, happy, neutral) separated by one week. Order of emotion-induction conditions was fixed, and not counterbalanced because of known difficulties with the induction of emotions like fear in a laboratory setting. Emotion experiences elicited in the lab are not influenced by pre-existing affective states [45]. In previous experiments (e.g., [54]), we found that when participants enter the lab for the first time their salivary cortisol levels (indicator of psychological stress) were high (e.g., de Groot et al. [54], Figure 3C). As it can take more than 20–30 min for this level to decline to a “relaxed” state, being stressed on the first visit might confound the induction of happiness or neutral/calm emotional states, while at the same time, the fear induction would benefit from it. So, to maximize the chance of the fear induction being successful, we decided to administer it always first in the context of pre-existing high cortisol in initial donor sessions. For the inverse reason, the neutral condition was always last. At this point, we expected donors to have become sufficiently familiar with the procedure and the experimenter and that they would be fairly relaxed when they arrived. At the same time, because the first (fear induction when being alone) and the second session (happiness together with others) had been so different we also expected them to be still curious about what would happen in the third session to counteract the possible boredom.

During the second emotion induction, donors sat together in groups varying from 2–4 because the co-presence of others is contagious and heightens a positive experience. As stated above, inducing emotions in the lab can be difficult [45]. Rather than making an effort to keep all inductions similar on other domains (number of people being tested in a condition), we made efforts to design the induction so as to maximize the chances that the emotion induction would be successful.

#### 4.1.1. Emotion Induction

Each of the emotion induction procedures involved watching a series of movie-clips for approximately 30 min. In the fear condition participants watched the original version of nine English-spoken horror film clips [60]; film database codes: 7, 16, 28, 32, 38, 46, 50, 55, 66; see also de Groot et al. 1. In the happy condition, participants watched four scenes carefully selected scenes (“Bear Necessities”, The Jungle Book; a short movie of Kurt Kuenne Validation; a comical opera scene from the Intouchables; and a televised prank on a company called Mobistar from the television programme Basta). In the neutral condition participants watched film clips that were effective in maintaining a calm (pleasant-neutral) state 1, such as a scene from the TV show “Rail Away.” The neutral video induced a pleasant, relaxing form of neutrality rather than plain neutrality, which could result in negative feeling states such as boredom or irritation [45]. The movie clips used in this study have been made publicly available on: https://osf.io/v9uf5/ (see Materials > Film clips.zip).

#### 4.1.2. Manipulation Check

First, participants dichotomously rated their experience of 20 different emotional states (yes/no). If they answered “no,” participants were scored a 0 on that state. If they answered “yes,” participants additionally rated their experience of the respective state on a separate 5-point scale ranging from 1 (a little bit) to 5 (extreme). The yes/no queries were used to discourage participants from treating a unipolar measure of a state as a bipolar measure of one emotion (e.g., sadness) and its polar opposite (e.g., happiness) [61]. The questionnaire included all 16 items from Russell’s [46] circumplex model (e.g., happy, sad) and four additional discrete emotion terms, namely surprise, anger, fear, and disgust. Given the happy, fear, and neutral emotion induction condition, the discrete emotions happiness and fear, but also calmness, were marked as “target states” and these were analyzed separately. Furthermore, based on the circumplex model of emotion [46], different arrangements of feeling states were averaged to create indicators of high arousal (alert, excited, elated, happy, tensed, nervous, stressed, upset), low arousal (sad, depressed, lethargic, fatigued, calm, relaxed, serene, contented), positive valence (alert, excited, elated, happy, contented, serene, relaxed, calm), and negative valence (sad, depressed, lethargic, fatigued, upset, stressed, nervous, tense).

Arousal and valence were also measured directly via the affect grid [47] —a 7 × 7 matrix, where valence (negative-positive) was represented horizontally, and arousal (high-low) vertically. Participants had to place an “X” in one of the squares, yielding two scores (valence, arousal) ranging from 1 to 7.

#### 4.1.3. Sweat Donation and Storage

Participants wore pre-cleaned t-shirts with absorbent pads attached via stainless steel poppets, 4 for each armpit. Following each session, pads from both the left and right underarm (*n* = 144 in total: 24 × 3 conditions × 2 axillae) were removed from the *t*-shirts using tweezers and scissors precleaned in a 96% ethanol solution. The pads were placed in labelled bags and vacuum-sealed, and bags were thereupon immediately stored in a freezer at −28 °C (Utrecht University, Utrecht, the Netherlands). After completion of the collection the samples were sent on dry ice by DHL same day delivery to URDV where they were stored at −80 °C.

### 4.2. Sweat Analysis Procedure with GC×GC-TOFMS

#### 4.2.1. Instrumentation

A Pegasus^®^ 4D GC×GC-TOFMS system (LECO) equipped with a liquid nitrogen cryogenic quad jet modulator and secondary oven was used for the sample analysis. The thermal desorption (TD) was performed with a thermal desorption unit (TDU) equipped with a MPS 2 auto-sampler and a liquid nitrogen (LN2) cooled CIS 4 programmed temperature vaporization (PTV) inlet (all from Gerstel) installed on an Agilent 6890 gas chromatograph (for further reference to methods used see Harker et al. [62]).

#### 4.2.2. Sample Preparation/Extraction

Pads were analyzed using the HeadSpace Sorptive Extraction (HSSE) technique. For the extraction, 250 mL Scott-Duran GLS80 bottles were used, with screw caps with PFTE septum. A small magnet was attached to the outside wall of the bottle, just below the neck of the bottle, using adhesive tape. Then the pad was transferred into the bottle using a clean pair of tweezers and 5 µL internal standard solution was added using a 5 µL transfer pipettor at the bottom of the bottle. Finally, a PDMS stir bar (1 cm × 1 mm; Gerstel) was attached with forceps to the inside bottle wall (attached to the magnet) and the bottle was closed with the screw cap with PFTE septum. Extraction was done in an oven at 60 °C for 2 h. After the extraction the stir bar was removed with forceps and placed in a clean glass thermal desorption liner. The glass liner was placed in the Twister tray of the MPS sampler, ready for analysis.

Reconditioning of stir bars was done after use by soaking in ultrapure water and methanol 1:1 (v/v) for 1–2 h each; stir bars were then removed from the solvent and dried on a clean surface at room temperature for 1 h. Finally, the stir bars were thermally conditioned in the glass thermal desorption liners for 30 min at 250 °C in a flow of helium using the Gerstel tube conditioner (TC) and stored in the MPS-2 Twister tray. Used bottles and caps were rinsed with water and methanol and finally thermally cleaned in an oven at 75 °C for approximately 8 h.

The internal standard solution contained 30.4 µg/mL five times deuterium labelled benzaldehyde (Aldrich) using methanol as the solvent. The peak area of the internal standard was only used to monitor the efficiency of the extraction and the stability of the instrument.

Per day/sequence all pads from two participants were measured. The participants were randomly selected. All six pads from one participant were measured after each other and the left and right axilla of one treatment were analyzed together (L&R, R&L, L&R). Finally the order of the treatment was varied (H, N,F & N,H,F or F,N,H & H, F,N). Each sequence was started with a blank tube and at the end of the sequence a defrost cycle of the modulator was applied.

#### 4.2.3. Thermal Desorption (TD) – GC×GC TOF MS

The stir bars were thermally desorbed in splitless mode by programming the TDU from 35 °C (held for 1 min) to 250 °C (held for 5 min) at 720 °C min^−1^ with 75 mL min^−1^ desorption flow. Desorbed compounds were focused at −120 °C on a liner filled with deactivated quartzwool in the LN2 cooled PTV inlet for subsequent GC×GC–TOF MS analysis. After desorption, the PTV inlet was programmed from −120 °C to 250 °C (held for 5 min) at 12 °C/sec to inject trapped compounds onto the analytical column. The injection was performed in split mode with a split ratio of 1:50. The first dimension (1D) column was a 30 m × 0.25 mm, df = 0.5 µm non-polar Rxi-5MS (Restek Corporation) and the second dimension (2D) column was 2 m × 0.15 mm, df = 0.15 µm mid-polar RTX-200 (Restek Corporation). Deactivated universal presstight connectors (Restek Corporation) were used for connecting the 1D and 2D columns. Helium was used as the carrier gas at a constant flow of 1.5 mL/min. The 1D GC oven temperature was initially set at 40 °C and held for 2 min, before being increased at 4 °C/min to 250 °C where it was held for 15 min. The temperature offset for the secondary oven was +10 °C relative to the 1D GC oven. The modulation period (PM) was 3 s, with a hot pulse time of 0.9 s and a cold pulse time of 0.60 s on each jet. The modulator temperature offset was +15 °C relative to the secondary oven. The transfer line was held at 275 °C. The TOFMS was operated in electron ionization (EI) mode at 70 eV, with an acquisition mass range of 35–300 amu, an acquisition rate of 150 Hz, and a detector voltage of 1800 V. The ion source was heated to 200 °C. Mass calibration and tuning was performed using perfluorotributylamine (PFTBA). An acquisition delay of 3 min was applied.

#### 4.2.4. Data Processing

**Figure A3:**
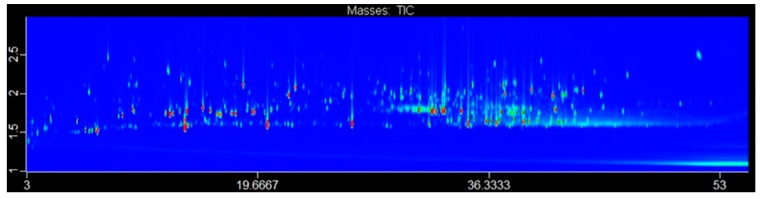
Example of a typical comprehensive GC×GC TOF MS contour plot of a test person, with x-axis denoting first dimension rt time in min (on non-polar column in Table A1), and y-axis denoting second dimension rt time is seconds (mid polar column in Table A1).

Data processing was performed using ChromaTOF^®^ (version 4.50; LECO, St Joseph, MI, USA). This software was used for peak finding, with each peak defined as being a unique feature corresponding to a unique deconvoluted combination of retention times and mass spectrum, mass deconvolution, integration, and library searching. The baseline offset was set to 1, just above the noise level and automatic smoothing was applied. The first dimension peak width was set to 10 s, while the second dimension peak width was set to 0.1 s. A 65% mass spectral match was required to combine the subpeaks with each other and their corresponding base peak. A minimum signal-to-noise ratio (S/N) of 100 was used for the base peak detection with a minimum of 2 apexing masses and sub-peak detection was cut-off at a minimum S/N of 10. Traditional, not adaptive, integration was used. Peak identification was performed by a forward search to the 2011 National Institute of Standards and Technology (NIST) mass spectral library database with a minimum similarity match of >80%. The Statistical Compare software feature within ChromaTOF^®^ was subsequently used to perform the peak alignment between samples using a mass spectral match threshold of 60%. Samples were input into the Statistical Compare and separated into different classes: Neutral (*n* = 48), Fear (*n* = 47, Happy (*n* = 47), Room (*n* = 24; denoted as Reference), and Clean Pads (*n* = 4). One pad of the Happy and Fear classes were missing, respectively. For the QC samples (clean pads spiked with deuterium labeled internal standards) a separate SC table was created. During the alignment, peak researching was performed with a minimum S/N cut-off of 50 in order to search for the peaks not found during the initial peak-finding step. A maximum retention time difference of 3 s (i.e., 1 modulation period) in the 1D and 0.05 s in the 2D was permitted during alignment to allow for retention time deviations between samples. During alignment, analytes were only retained if found in at least three samples OR if found in 50% of the samples within a class. The resulting peak table (3796 peaks) was exported and used for further multivariate analysis. Figure A3 (Appendix A) shows an example of a typical comprehensive GC×GC TOF MS contour plot of a test person.

**Figure A4:**
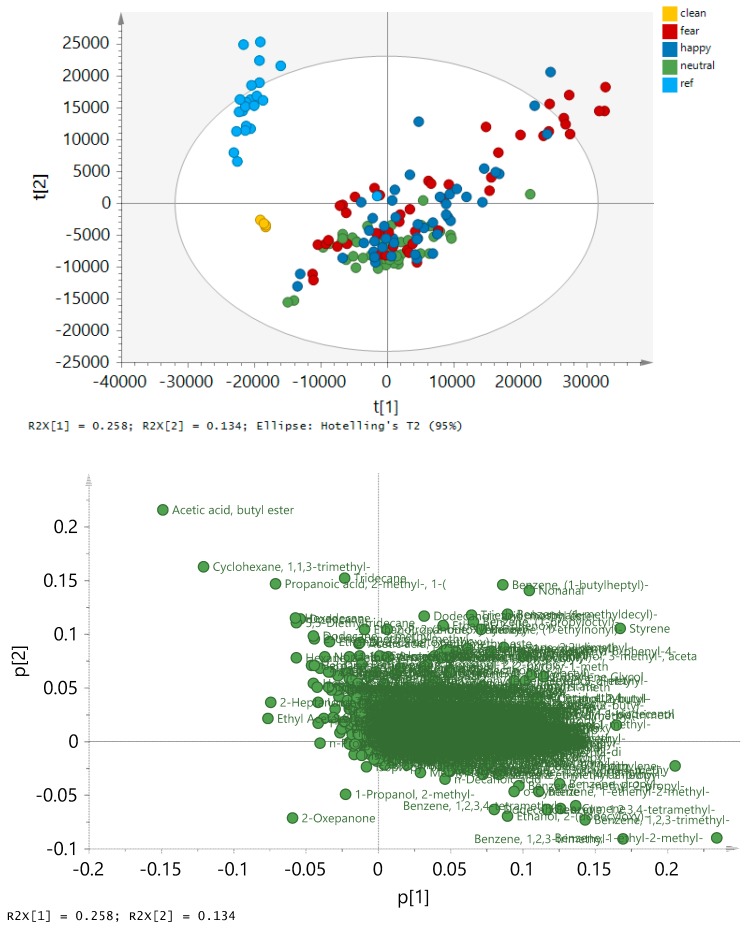
PCA performed on 3796 RT/MS peaks and 169 pads using Pareto scaling. The scores plot (upper panel) displays the variation of the pads classified as clean (from clean pads), reference (from pads deposited in the room), and the condition samples (Neutral, Happy, Fear from the subjects). The loading plot (lower panel) displays the variation of the analytes with tentative assignments.

The statistical analysis was performed in SIMCA (version 15.02.5959, Sartorius Stedim Biotech, Umëa, Sweden). Initially, principal component analysis (PCA) was performed on all 3796 peaks and pads (141 condition samples +24 reference samples + 4 clean samples = 169) to distinguish between reference and clean pads from the sweat pads from the subjects. Samples from left and right underarms of the same individual were treated as different samples. A visual inspection of PLS-DA score plots on all left panels of Figure 2 by donor ID and arm (L vs. R) distribution showed that L and R samples from the same donor were not identical but also that variation between underarms was smaller than between donors. The peak areas were scaled to Pareto prior to analysis [63]. This scaling method attenuates large peaks and enhances small peaks, and compromises between mean centering and unit variance scaling. This method was chosen to focus on the main effects. The scores plot (Figure A4; Appendix A) shows a clear separation between the control pads and the pads from the subjects, except for the one reference pad that was contaminated with sweat from a subject. Various (tentatively assigned) volatiles were detected in the control samples including for example acetic acid butyl ester, 1,1,3-trimethylcyclohexane, 1,1-dimethylehtyl-2-methyl-propionic acid, tricane, diethyltridecane, dodecane heptanone, hexadecane, 5,5-diethyltridecane, and ethylacetate. PCA analysis excluding the reference and clean samples identified one pad in the Happy group as outlier.

For the comparison between the condition classes the data were further cleaned up. For this, only peak intensities above an (arbitrary) threshold of 2400 were considered to focus on higher abundant peaks (23% of the dataset). Moreover, we excluded peaks (*n* = 2139) for which the median concentrations calculated over all classes were zero (defined as <2400) to focus on peaks that are representative for the conditions. Then, all zeros were removed, to prevent the interference of zero values, because they were not true values but rather indicated an absence of detection, small signals, or noise. In addition, the condition-matching pads of the missing pads (*n* = 6) were excluded to have for each subject and each arm the same number of condition pads. In total, 135 pads including 45 pads per condition class and 1655 peaks were taken to further analysis.

To identify the peaks that discriminate between the condition classes, partial least squares discriminant analysis (PLS-DA) was performed. This supervised method allows for finding the maximum differences between the conditions (rather than the maximum variance as in case of PCA). In total, three PLS-DA models were calculated, namely Neutral versus Happy, Neutral versus Fear, Happy versus Fear. The data were not normalized and Pareto scaling was applied prior to analysis. All models were validated using seven-fold cross-validation and permutation testing with 900 models of randomly permuted data [64]. Q^2^-values were calculated to assess the predictive performance of the models. A Q2 value of 1 indicates maximum predictive power, whereas Q^2^ values close to or below 0 indicate a lack of predictive power.

To visualize the final variances of all three conditions, PCA was performed on 94 peaks that were in total found to be discriminative in the three PLS-DA models. The data were not normalized and Pareto scaling was applied prior to analysis.

Univariate statistical analysis was performed in JMP^®^ Pro 12.0.1. (SAS Institute Inc. 2013, 198 Cary, NC, USA). Receiver operating characteristic (ROC) curves were plotted to additionally confirm the discriminative power of each individual discriminative peak identified from the PLS-DA models Neutral-Fear, Neutral-Happy, Fear-Happy. The ROC curve displays the true positive rate (TPR; probability of correct classification, sensitivity) versus the false positive rate (FPR; probability of wrong classification, 1-specificity) based on the ranked peak areas against the binary classifier system (condition I e.g., Neutral versus condition II e.g., Fear). The condition class with the higher concentrations was defined as the positive level. Peak areas from condition I are perfectly discriminated from condition II, if they are systematically higher (or lower) in condition I than in condition II. In this case, ROC curve passes through the upper left corner and the area under the ROC curve (AUROC) is maximal. The steepness of the ROC curve indicates the relation between the true positive rate and the false positive rate. A peak is maximally discriminative when the true positive rate is maximum and the false positive rate is minimum.

Box plots were made on the discriminative peaks from the PLS-DA models and on the sum of all peak areas to visualize differences between the three condition groups. The box plot displays the maximum, 75 quartile, median, 25 quartile, and minimum values. In addition, the peak areas of the individual peaks were plotted against the total intensities to assess whether condition-dependent changes of the peaks are related to the total sweat production.

### 4.3. Ethics Statement

The study protocol was approved by the Wageningen University ethics committee in 2014 (U-IEC dossier 14/23; approval date 30 September 2014). All methods were performed in accordance with national and international guidelines and regulations, and with the Helsinki Declaration of 1975, as revised in 2013 [65]. Written informed consent was obtained from the sweat donors prior to starting the study. Sweat donors received a financial remuneration of €50, for participating in the study.

## Figures and Tables

**Table 1:** Results of emotion manipulation on 20 discrete emotion states, three of which are the target states, rated from 0 to 5, and separately on valence, and arousal (affect grid).

Feeling	Friedman Test	Comparison Condition (Intended Emotions)			Order
		Fear vs. Happy	Fear vs. Neutral	Happy vs. Neutral	
*Target State*					
Fear	c^2^ = 38.00, *p* < 0.001	***Z* = 3.9, *p* < 0.001**	***Z* = 3.9, *p* < 0.001**	*Z* = 0.0, *p* > 0.999	F>H=N
Happiness	c^2^ = 40.77, *p* < *0*.001	***Z* = −4.5, *p* < 0.001**	*Z* = −2.4, *p* = *0*.017	***Z* = 4.2, *p* < 0.001**	H>N>F
Calmness	c^2^ = 33.11, *p* < *0*.001	***Z* = −3.9, *p* < 0.001**	***Z* = −4.0, *p* < 0.001**	*Z* = −1.7, *p* = 0.096	N=H>F
*Affect Grid*					
Valence	c^2^ = 34.63, *p* < 0.001	***Z* = −4.2, *p* < 0.001**	***Z* = −3.2, *p*< 0.001**	***Z* = 3.8, *p* < 0.001**	H>N>F
Arousal	c^2^ = 31.73, *p* < 0.001	*Z* = 2.6, *p* < 0.001	***Z* = 4.3, *p*< 0.001**	***Z* = 3.2, *p* < 0.001**	F>H>N
*Other States*					
Tense	c^2^ = 41.38, *p* < 0.001	*Z* = 4.1, *p* < 0.001	*Z* = 4.1, *p* < 0.001	*Z* = −1.0, *p* = 0.317	F>N=H
Nervous	c^2^ = 40.00, *p* < 0.001	*Z* = 4.0, *p* < 0.001	*Z* = 4.0, *p* < 0.001	*Z* = 0.0, *p* > 0.999	F>H=N
Relaxed	c^2^ = 39.74, *p* < 0.001	*Z* = −4.3, *p* < 0.001	*Z* = −4.2, *p* < 0.001	*Z* = 1.5, *p* = 0.137	H=N>F
Alert	c^2^ = 39.00, *p* < 0.001	*Z* = 4.1, *p* < 0.001	*Z* = 4.2, *p* < 0.001	*Z* = 1.8, *p* = 0.061	F>H=N
Elated	c^2^ = 36.90, *p* < 0.001	*Z* = −4.2, *p* < 0.001	*Z* = −1.5, *p* = 0.141	*Z* = 4.0, *p* < 0.001	H>N=F
Serene	c^2^ = 30.90, *p* < 0.001	*Z* = −3.8, *p* < 0.001	*Z* = −4.1, *p* < 0.001	*Z* = −1.2, *p* = 0.213	N=H>F
Content	c^2^ = 29.54, *p* < 0.001	*Z* = −4.0, *p* < 0.001	*Z* = −2.0, *p* = 0.050	*Z* = 3.6, *p* < 0.001	H>N>F
Disgust	c^2^ = 29.39, *p* < 0.001	*Z* = 3.4, *p* < 0.001	*Z* = 3.4, *p* < 0.001	*Z* = −1.0, *p* = 0.317	F>N=H
Surprised	c^2^ = 27.49, *p* < 0.001	*Z* = 3.3, *p* < 0.001	*Z* = 3.7, *p* < 0.001	*Z* = 2.6, *p* = 0.011	F>H>N
Fatigued	c^2^ = 20.35, *p* < 0.001	*Z* = 0.3, *p* = 0.752	*Z* = −3.5, *p* < 0.001	*Z* = −3.2, *p* < 0.001	N > H=F
Stressed	c^2^ = 17.43, *p* < 0.001	*Z* = 2.7, *p* = 0.007	*Z* = 2.7, *p* = 0.007	*Z* = −1.0, *p* = 0.317	F>N=H
Upset	c^2^ = 16.29, *p* < 0.001	*Z* = 2.9, *p* = 0.004	*Z* = 2.9, *p* = 0.004	*Z* = −1.4, *p* = 0.157	F>N=H
Lethargic	c^2^ = 11.35, *p* = 0.003	*Z* = −0.5, *p* = 0.593	*Z* = −1.9, *p* = 0.062	*Z* = −2.2, *p* = 0.030	N>H=F
Excited	c^2^ = 11.15, *p* = 0.004	*Z* = 0.0, *p* = 0.972	*Z* = 3.0, *p* = 0.002	*Z* = 2.9, *p* = 0.004	F=H>N
Depressed	c^2^ = 4.00, *p* = 0.135	*Z* = 1.4, *p* = 0.157	*Z* = 0.1, *p* = 0.916	*Z* = 1.8, *p* = 0.068	F=H=N
Sad	c^2^ = 2.00, *p* = 0.368	*Z* = 1.0, *p* = 0.317	*Z* = −0.6, *p* = 0.564	*Z* = −1.4, *p* = 0.157	F=H=N
Angry	c^2^ = 1.40, *p* = 0.497	*Z* = 0.3, *p = 0*.785	*Z* = 1.4, *p* = 0.157	*Z* = 0.5, *p* = 0.655	F=H=N

Note: Left column: results from non-parametric Friedman test, degrees of freedom: 2, *n* = 24. Middle columns: follow-up Wilcoxon signed-ranks tests for specific comparisons. Right column: expected ordering of conditions on a particular self-report variable, with signs (=, <, >) indicating relative effect direction. Manipulation checks in bold.

**Table 2:** Response pattern (high vs. low) of the two chemical compound classes by emotion condition (neutral, fear, and happy) with happy showing a bipolar pattern with individuals in subcluster HF overlapping with Fear pattern; in subcluster HN overlapping with Neutral. *n* = number of donors.

Chemical Class		Condition		
	Neutral	Fear	Happy	Subcluster
Esters and 5-rings	High	Low	Low	HF (*n* = 14)
			High	HN (*n* = 8)
Linear aldehydes/ketones	Low	High	High	HF (*n* = 14)
			Low	HN (*n* = 8)

**Table 3:** Significant compounds by class 1 and class 2 compounds using Tukey–Kramer, all pairs testing with *p* < 0.05.

Chemical Class		
**-**	**CLASS 1**	**CLASS 2**
	Linear Aldehydes / Ketones / Alcohols	Esters and Some Ring Structures *
1	Peak 146: 3-penten-2-one	Peak 37: ethyl acetate
2	Peak 234: hexanal	Peak 61: 4-methyl-1,3-dioxolane
3	Peak 505: 2-heptenal	Peak 122: propyl acetate
4	Peak 560: 1-octen-3-ol	Peak 161: 1-ethoxy-2-propanol
5	Peak 638: octanal	Peak 251: butyl acetate
6	Peak 662: 2,4-heptadienal	Peak 531: 2-cyclopenten-1-one, 3-methyl
7	Peak 794: 2-octenal	Peak 2360: 1,6-dioxacyclododecane-7,12-dione
8	(Peak 957: nonanal)	
9	Peak 920: 2-hexylfuran	
10	Peak 979: octadienal	
11	Peak 1134: γ-heptyl-lacton	
12	(Peak 1296: decanal)	
13	Peak 1313: nonadienal	
14	Peak 1449: 2-Me-decanal	
15	Peak 1587: decadienal	
16	Peak 2127: 3-tridecanone	

Note: Compounds in parentheses are peaks that were identified with minor differences. * Not all ring structures are in this class.

## Supplementary Materials

The following are available online at: Movie clips used in this study: https://osf.io/v9uf5/ (see Materials > Film clips.zip); raw data etc., will be made available upon publication. The following are available online at https://www.mdpi.com/2218-1989/10/3/84/s1. Figure S1: Individual box plots for each peak across the emotion condition fear, happy, and emotionally neural, S2 A+B: Additional testing to explore bipolar pattern in happy chemical cluster: results + statistical analyses, S3: Study to address the social factor during happiness induction, Figure S3: Scatter plot of self-reported emotions in happiness condition (top panel) and neutral (bottom panel) on PC1 and PC2 comparing scores from four studies (*n* = 84) in which multiple men sat together and happiness condition was always second (pink) vs. a study (*n* = 24) in which men were sitting alone and condition was counterbalanced (with happiness condition being first or second; turquoise).

## Appendix A. Additional Data Figures and Tables

**Table A1:** Discriminative peaks from three PLS-Da models with peak in order of first dimension retention time on non-polar column (RT Dim 1) with addition of second dimension retention time on mid-polar column (RT Dim 2).

Peak	RT Dim 1	RT Dim 2	Mass	Discrimination	Up or Down
37	213.065	1.50373	43	F H vs N	D
61	252.986	1.54448	87	F H vs N	D
75	266.896	1.69866	84	F H vs N	D
82	278.881	1.52216	87	F H vs N	D
94	290.867	1.5497	87	F H vs N	D
122	311.842	1.70207	61	F H vs N	D
146	347.798	2.07133	69	F H vs N	U
161	368.059	1.68569	59	F H vs N	D
177	381.986	2.01085	59	F H vs N	D
199	410.852	1.84624	45	F H vs N	U
211	422.849	2.59792	43	F H vs N	D
221	431.698	2.17416	55	F H vs N	U
234	446.54	1.94374	56	F H vs N	U
235	445.611	2.1115	45	F H vs N	D
251	470.651	1.85195	43	F H vs N	D
299	536.554	1.81135	45	F H vs N	D
456	713.831	1.96898	61	F H vs N	D
505	773.287	2.35098	83	F H vs N	U
531	794.262	2.88303	96	F H vs N	D
560	824.226	1.74228	57	F H vs N	U
562	823.532	1.91573	103	H vs F N	U
638	878.161	2.06622	57	F H vs N	U
662	896.75	2.4186	81	F H vs N	U
747	967.754	2.2181	146	F H vs N	U
760	979.795	2.2298	91	H vs F N	U
783	1001.01	0.424779	85	F H vs N	U
794	1007.01	2.33448	70	F H vs N	U
799	1010	2.30056	99	F H vs N	U
841	1038.33	2.20129	59	F H vs N	U
916	1084.9	1.57542	93	H vs F N	D
920	1084.97	1.65668	81	F H vs N	U
957	1111.88	2.04592	57	F H vs N	U
965	1118.6	1.60533	267	F H vs N	D
979	1123.87	2.38479	81	F H vs N	U
1021	1146.78	2.91127	87	F H vs N	D
1037	1150.08	2.73321	99	F H vs N	U
1134	1225.92	0.285066	85	F H vs N	U
1166	1240.73	2.2519	113	F H vs N	U
1185	1252.26	1.75714	207	H vs F N	U
1206	1263.01	2.21667	101	F H vs N	D
1296	1336.58	2.01166	57	F H vs N	U
1313	1354.66	2.32802	81	F H vs N	U
1367	1411.52	1.83798	59	F H vs N	U
1387	1422.39	1.84604	59	F H vs N	U
1405	1433.16	0.157468	113	H vs F N	U
1437	1458.51	1.54951	163	F H vs N	U
1449	1462.46	1.9767	58	F H vs N	U
1450	1462.46	2.1945	127	F H vs N	U
1457	1465.46	2.42029	131	F H vs N	U
1497	1507.46	2.15058	59	H vs F N	U
1521	1525.41	1.82177	207	H vs F N	U
1553	1549.82	2.17105	59	F H vs N	D
1562	1555.15	1.69778	281	H vs F N	U
1587	1570.33	2.26863	81	F H vs N	U
1599	1581.97	1.54985	73	H vs F N	U
1616	1591.93	1.74071	59	F H vs N	U
1656	1628.35	0.0582741	84	F H vs N	U
1672	1639.32	1.84592	59	F H vs N	U
1673	1638.78	2.08221	133	H vs F N	U
1735	1687.01	1.5716	71	F vs H N	U
1765	1711.21	1.71539	69	F vs H N	U
1776	1717.15	1.82356	59	F H vs N	U
1780	1723.15	1.93293	114	F H vs N	U
1810	1741.73	2.28162	59	F H vs N	D
1842	1762.1	1.84815	109	H vs F N	U
1850	1767.88	2.30028	87	F H vs N	D
1866	1777.23	2.70362	87	F H vs N	D
1911	1804.52	1.54262	57	H vs F N	U
2011	1848.74	1.93918	95	F H vs N	U
2019	1848.07	1.73778	97	H vs F N	D
2050	1867.03	1.65334	92	H vs F N	U
2073	1875.98	2.19588	177	F H vs N	U
2088	1884.55	1.7368	70	F vs H N	U
2107	1893.49	1.5439	70	F vs H N	U
2127	1902.93	1.91097	72	F H vs N	U
2149	1913.33	1.96941	58	H vs F N	U
2209	1944.88	1.68335	191	F H vs N	D
2262	1974.91	1.79818	59	H vs F N	U
2277	1980.84	1.72275	57	F H vs N	U
2348	2013.6	2.31272	87	F H vs N	D
2360	2022.79	2.48686	100	F H vs N	D
2412	2046.83	1.84665	113	F H vs N	U
2490	2091.72	1.88072	114	F H vs N	U
2529	2112.93	1.62908	119	H vs F N	D
2578	2139.68	1.87183	150	F vs H N	U
2608	2156.88	1.76811	70	H vs F N	D
2625	2165.99	1.58243	91	F H vs N	U
2756	2220.55	1.86757	58	F H vs N	U
3100	2391.31	2.21692	219	F H vs N	U
3190	2457.32	1.60417	151	H vs F N	U
3231	2493.19	1.84005	69	F vs H N	U
3234	2496.29	1.60178	151	H vs F N	U
3437	2739.03	1.59881	151	H vs F N	U
3489	2830.62	1.62092	149	H vs F N	U

Note: “Mass” corresponds to the unique ion in the deconvoluted EI spectrum determined by the LECO ChromaToF software. “Discrimination” indicates significance from either of Happy (H) and Fear (F), or both, over Neutral (N) associated with donor peaks being up (U) or down (D) for Happy/Fearful.
