# Supplementary material for: Chemical Fingerprints of Emotional Body Odor
Source: Metabolites. 2020 Feb 28;10(3):84. doi: 10.3390/metabo10030084 (PMC7142800; doi:10.3390/metabo10030084)
Supplement: Supplementary file 1 [file metabolites-10-00084-s001.pdf]

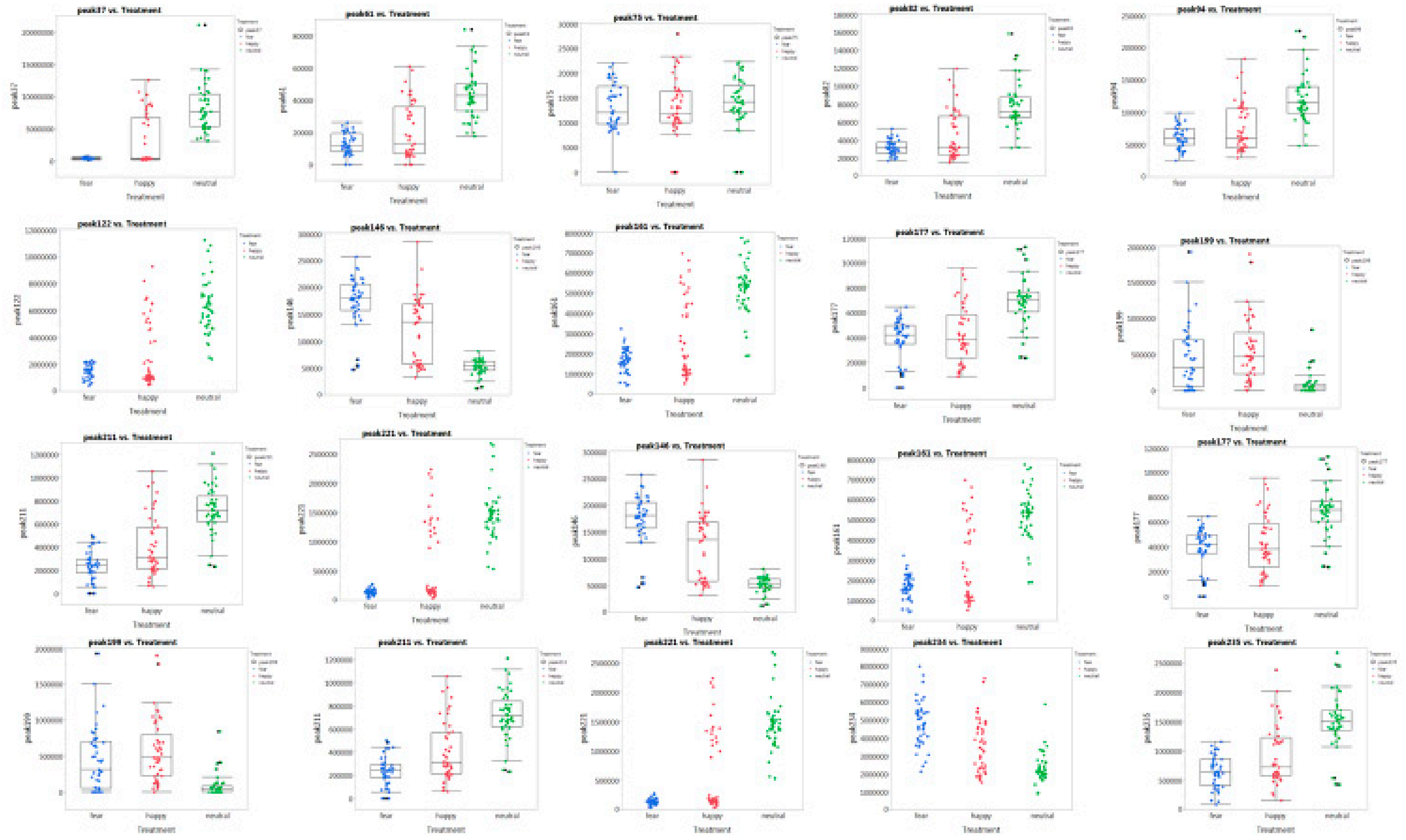
peak37 vs. Treatment
peak61 vs. Treatment
peak75 vs. Treatment
peak82 vs. Treatment
peak94 vs. Treatment
peak122 vs. Treatment
peak146 vs. Treatment
peak161 vs. Treatment
peak177 vs. Treatment
peak199 vs. Treatment
peak211 vs. Treatment
peak221 vs. Treatment
peak146 vs. Treatment
peak161 vs. Treatment
peak177 vs. Treatment
peak199 vs. Treatment
peak211 vs. Treatment
peak221 vs. Treatment
fear
happy
neutral
Treatment

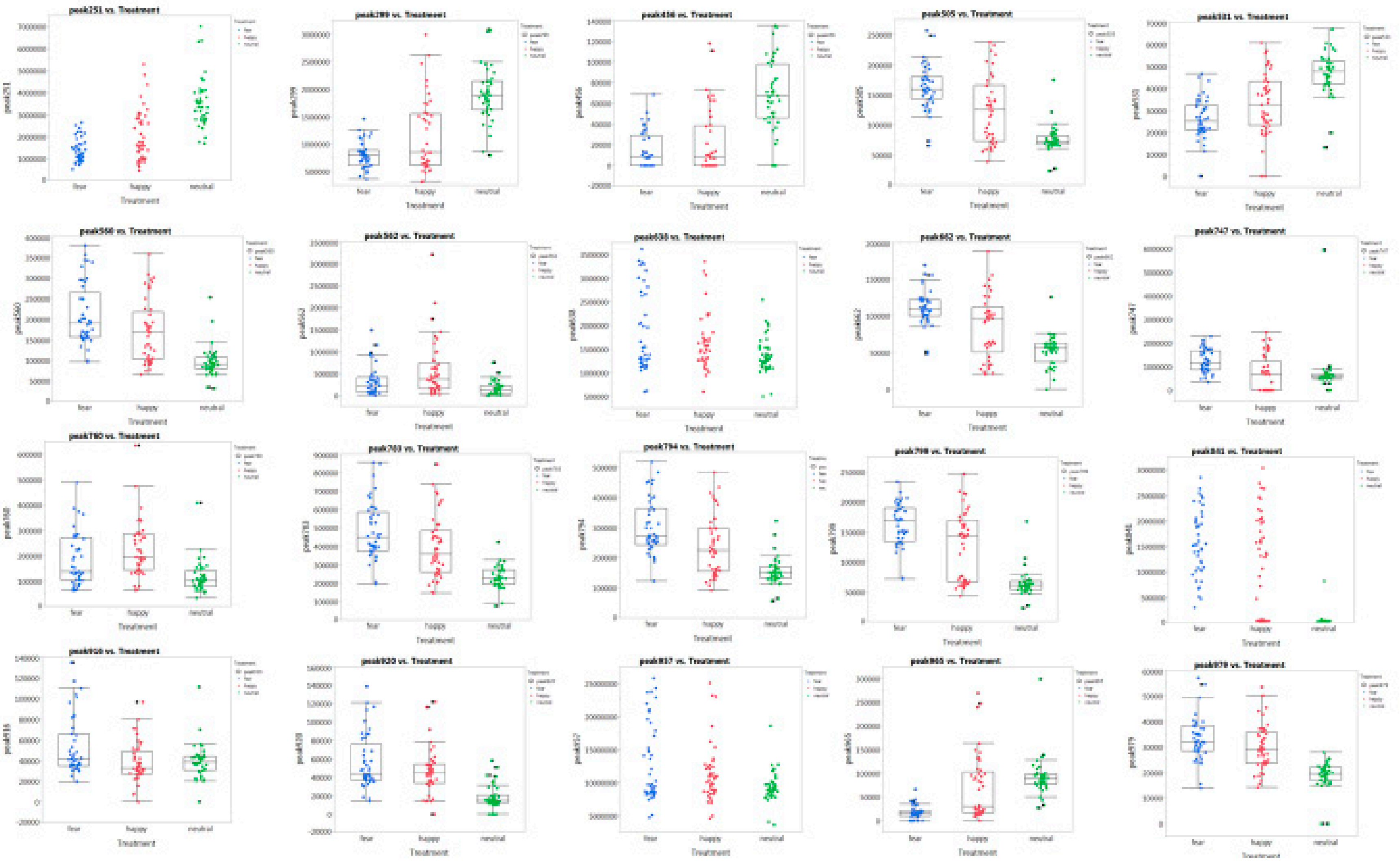
peak251 vs. Treatment
Treatment
fear
happy
neutral

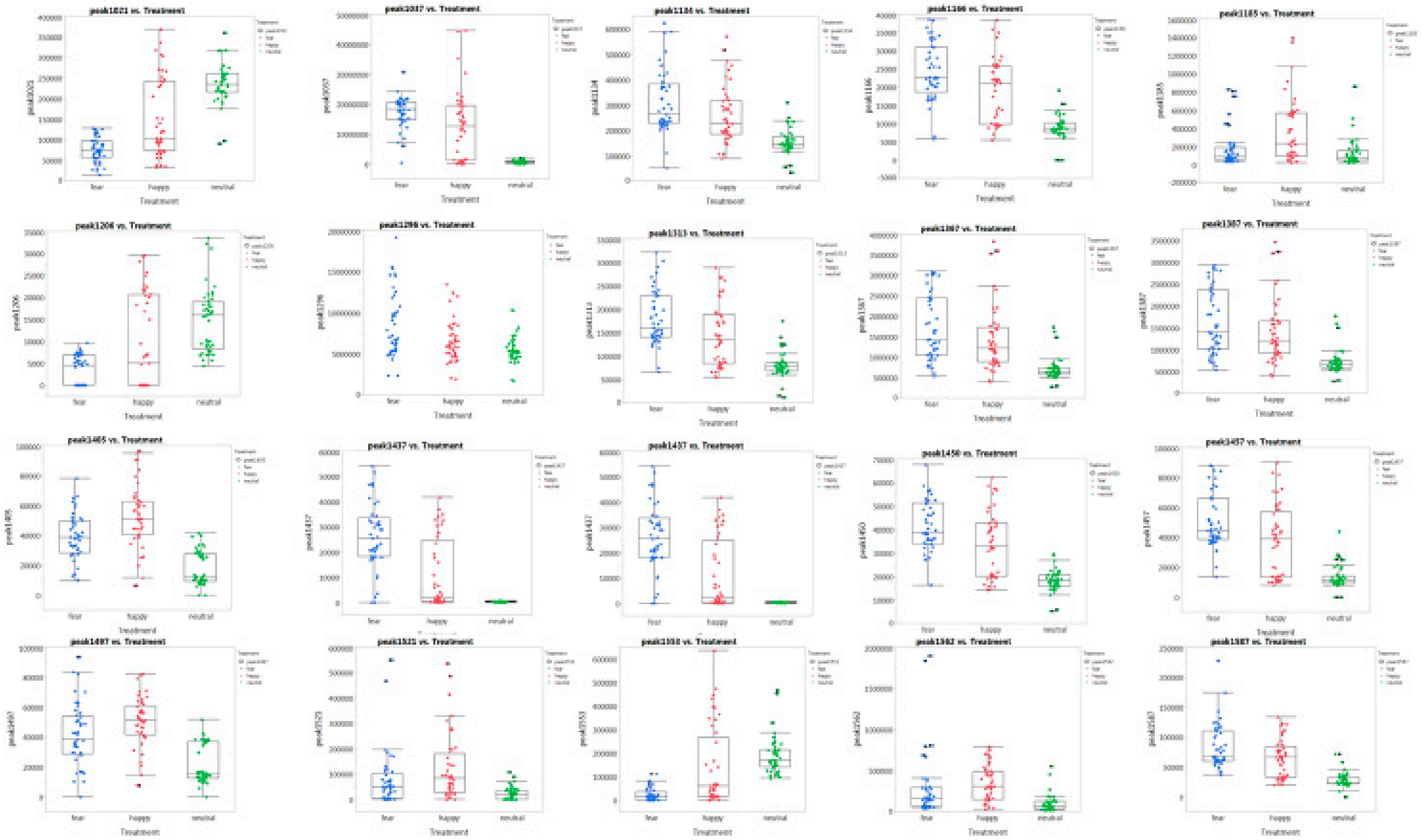
fear
happy
neutral
Treatment

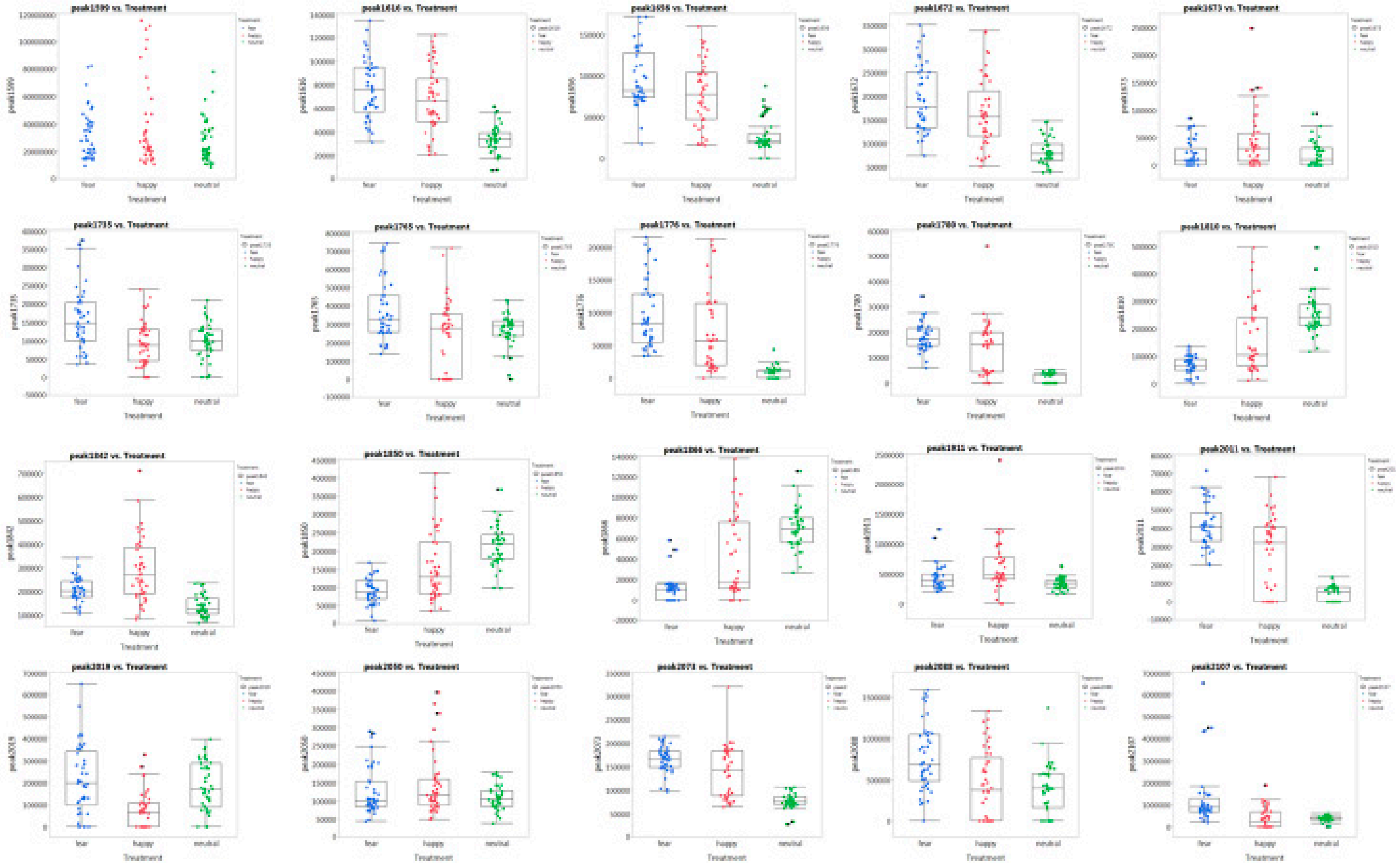

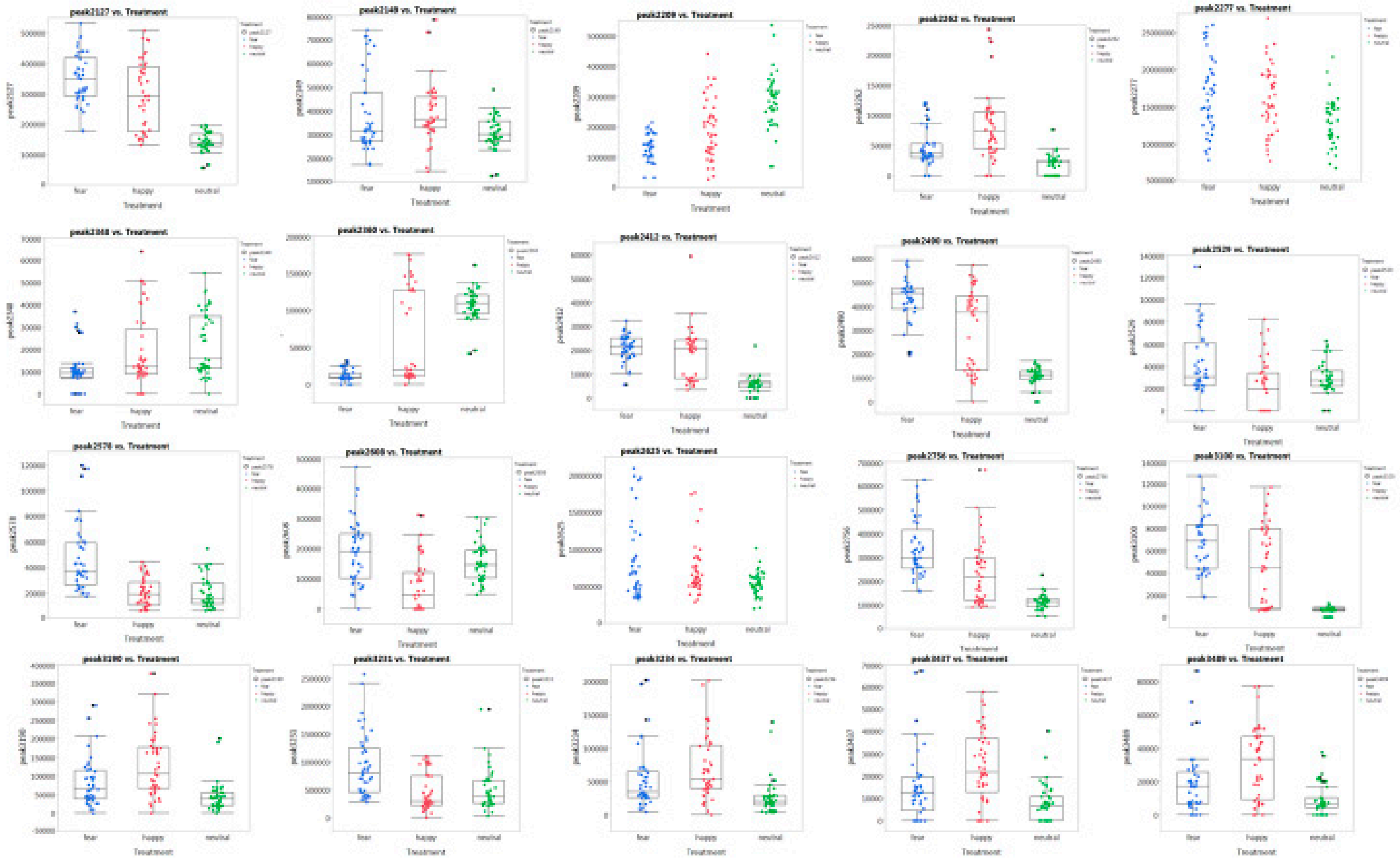

Figure S1. Individual box plots for each peak across the emotion condition fear, happy and emotionally neural, supplementary to Figures 2 and 3.

S2A. Additional testing to explore bipolar pattern in happy chemical cluster: results

The HF chemical sub-cluster consisted of 14 donors, the HN sub-cluster of 8, and 2 donors' chemical profiles who fit neither, and were excluded from analysis. A logistic regression with cross-validation and regularization including emotion self-report data was used to find which emotional states best distinguish between the chemical clusters. As described in the data analysis plan as Step 1, data was randomly split from each participant into training and test subsets. Following that, logistic regression combined with regularization and cross-validation was fitted on the training dataset, in order to find models that can distinguish between neutral and fear conditions, can be applied to new data, and are as simple as they can be. After finding the models which performed best in the training set, their accuracy was tested using the test subset.

Two models resulted from the training subset. The simplest model only needed the variable alert to distinguish between the conditions. The more complex model used serene, alert, lethargic, fatigued, relaxed, and tensed to distinguish between the conditions. The predictive accuracy of the two models was then assessed on the test set. The simplest model, using only alert, had 90.5% accuracy on the test set. The more complex model had an accuracy of 92.9%. Since the benefits of the more complex model were not good enough to justify such a high number of variables, only the simplest model, which relied on alert, was kept for distinguishing between chemical clusters.

Before using alert to predict chemical clustering data, the distribution of alert across the two chemical clusters was visualized. Alertness was low, and indistinguishable between the two chemical clusters (MHF = 0.64, MHN = 1.13, $t(20) = -0.71$, $p = 0.48$), and, whereas it was much higher in fear condition, compared to neutral condition (difference = 3.14, 95% CI = 2.81, 3.48, d = 2.04, $t(83) = 18.7$, $p < 0.001$) . This made it clear that the model established earlier could not perform well on this data, since it would require the 'neutral' cluster to be much lower than the 'fear' cluster on alert. Therefore, this model received any further attention.

A second cross-validated and regularized logistic regression model which relied on chemical clustering data suggested that content and serene are best used for distinguishing the two chemical clusters in the happy group with content being the best predictor. Together, they can distinguish clusters with 91% accuracy. Similarity in the patterns of serene and content between clusters and conditions was assessed. While serene was much higher in the neutral experimental condition, compared to the fear experimental condition (difference = 2.64,. 95% CI = 1.93, 3.34, Cohen's d = 1.66), it was only insignificantly higher in the HF chemical sub-cluster, compared to the HN chemical sub-cluster (difference = 0.786 , 95% CI = -0.315, 1.89, Cohen's d = 0.66). Next, content was insignificantly higher for the neutral condition, compared to fear condition (difference = 0.864 , 95% CI = -0.07, 1.8, Cohen's d = 0.41), but was significantly higher in the HN sub-cluster, compared to HF sub-cluster (difference = 0.732 , 95% CI = 0.2, 1.26, Cohen's d = 1.28). Furthermore, to better understand the two chemical sub-clusters in the happy group, their average levels of happiness was assessed, which clearly distinguished happy condition from fear and neutral condition. Happiness was virtually indistinguishable in the chemical sub-clusters ($t(20) = 0.18$, $p = 0.86$, and very high on both(MHF = 3.79, MHN = 3.75). Taken together, with the findings regarding alert it appears that the chemical sub-clusters HF and HN for participants in happy condition do not follow the same emotional patterns as the actual neutral and fear conditions.

S2B. Additional testing to explore bipolar pattern in happy chemical cluster: statistical analyses

To assess correspondence between chemical clusters and experimental conditions, as a first step, a statistical model was built that can accurately and reliably distinguish between fear and neutral conditions. As a second step, this model was then used to distinguish between the HF and HN sub-clusters. The reasoning behind it was that if a model that can use emotional states to distinguish between emotion conditions can similarly distinguish between chemical clusters, then it follows that both experimental conditions and chemical clusters follow similar emotional patterns. In other words, donors who showed overlap in terms of chemical composition of sweat between happy and fearful conditions, possibly also showed such overlap in profile of pertinent emotional states experienced during happy and fearful conditions.

Cross-validated regularized models, optimal for doing an exploratory analysis that uses many variables and intended to generalize to new/unseen data [1], were used. Tuning parameters, as used in regularization, dictate how many variables can be used in a regression model, and how large the model's slopes can be [1]. Whereas cross-validation involves building a model using parts of the data, and testing it on the remaining data [1]. By combining cross-validation and regularization, it is possible to find optimal tuning parameters, which create models that generalize to unseen data. It is recommended to use two values of tuning parameters: one in which the model performs best on unseen data, and one which results in the simplest model whose accuracy is no more than 1 standard error from the most accurate model [1]. Models based on these optimal tuning parameters are then tested on the unused 'test' subset, and if they perform well, they can be said to accurately and reliably predict experimental conditions.

Only single-item emotional variables were used in the analysis, due to the results from PCA being inconclusive.

1. Step 1: As a first step in the analysis, cross-validated regularized logistic regression was used to distinguish between conditions using the different emotional states. Then, the models suggested by the regression were applied to the chemical clustering data. Data from fear and neutral conditions were divided into two subsets, with each subset holding one observation per participant – from one of the conditions – which was randomly assigned per subset. This was done to better approximate the statistical constraints of the chemical clustering data, where there is only one observation per participant. Then, using only one of the two subsets, the 'training set', a 10-fold cross validation was used to assess which tuning parameter is to be used for regularization. Following this, the most accurate and least complex models that were built using the training set were tested on the testing set. Those that performed well were then used to predict clustering data.

2. Step 2: Following model selection, the models that best distinguish between fear and neutral emotion conditions at the test subset were used to predict chemical sub-clusters HF and HN. If they did with high accuracy, then it was concluded that chemical clustering and experimental conditions follow similar emotional patterns. If they did not a cross-validated regularized logistic regression would be performed on the chemical clustering data. In that case the emotional patterns that distinguish chemical clusters were assessed using additional data on experimental conditions. If the affective patterns that distinguish between chemical clusters do not similarly distinguish between experimental conditions, and vice versa, then chemical clusters and experimental conditions do not follow the same emotional patterns.

[1]. James, G.; Witten, D.; Hastie, T.; Tibshirani, R. *An Introduction to Statistical Learning*; Springer Texts in Statistics; Springer New York: New York, NY, 2013; ISBN 978-1-4614-7137-0.

S3: Study to address social factor during happiness induction.

After the completion of the study reported in the main text, we conducted a separate study comparing self-reported emotions of 24 male donors who were individually seated in both the happiness and neutral condition, against the results from 84 male donors collated from 4 previous studies with 2-4 males seated together in the happiness condition. This was done to explore whether emotions experienced from emotion induction of happiness are different depending on whether males in the happy condition are seated alone or together. We also addressed another limitation in that study by counterbalancing condition order (happy first, neutral first). As can be seen in Figure S3 no differences were found between the new study and the combined data from the previous studies on self-reported emotion.

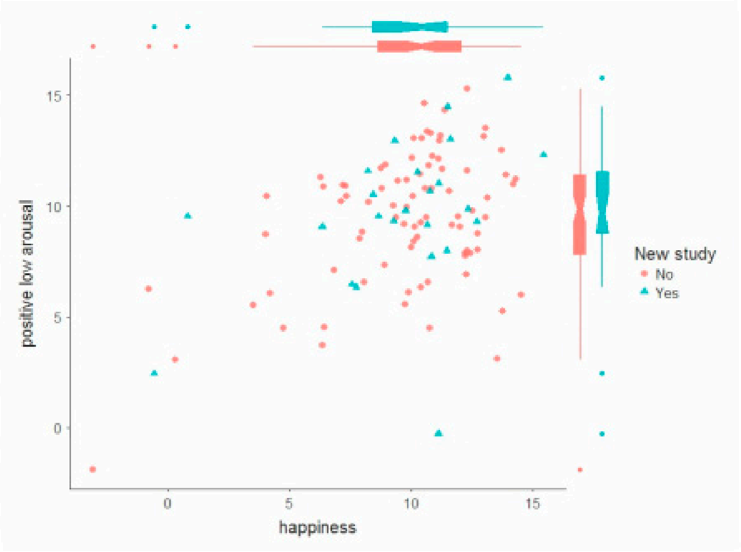

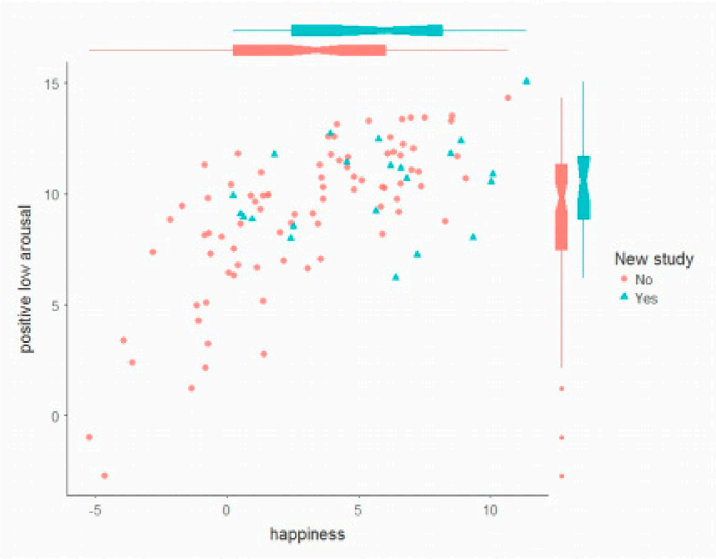

Figure S3. Scatter plot of self-reported emotions in happiness condition (top panel) and neutral (bottom panel) on PC1 and PC2 comparing scores from 4 studies (n=84) in which multiple men sat together and happiness condition was always second (pink) vs. a study (n=24) in which men were sitting alone and condition was counterbalanced (with happiness condition being first or second; turquoise).
